# CCR4 Controls the Suppressive Effects of Regulatory T Cells on Early and Late Events during Severe Sepsis

**DOI:** 10.1371/journal.pone.0133227

**Published:** 2015-07-21

**Authors:** Raphael Molinaro, Cyntia Pecli, Rafael F. Guilherme, José Carlos Alves-Filho, Fernando Q. Cunha, Claudio Canetti, Steven L. Kunkel, Marcelo T. Bozza, Claudia F. Benjamim

**Affiliations:** 1 Instituto de Ciências Biomédicas, Universidade Federal do Rio de Janeiro, Rio de Janeiro, RJ, Brazil; 2 Departamento de Farmacologia, Faculdade de Medicina de Ribeirão Preto, Universidade de São Paulo, Ribeirão Preto, SP, Brazil; 3 Instituto de Biofísica Carlos Chagas Filho, Universidade Federal do Rio de Janeiro, Rio de Janeiro, RJ, Brazil; 4 Department of Pathology, University of Michigan, Ann Arbor, Michigan, 48109, United States of America; 5 Departamento de Imunologia, Instituto de Microbiologia, Universidade Federal do Rio de Janeiro, Rio de Janeiro, RJ, Brazil; University Medical Center of the Johannes Gutenberg University of Mainz, GERMANY

## Abstract

Sepsis is a deadly disease characterized by an overwhelming release of inflammatory mediators and the activation of different types of cells. This altered state of cell activation, termed leukocyte reprogramming, contributes to patient outcome. However, the understanding of the process underlying sepsis and the role of regulatory T cells (Tregs) in sepsis remains to be elucidated. In this study, we investigated the role of CCR4, the CCL17/CCL22 chemokine receptor, in the innate and acquired immune responses during severe sepsis and the role of Tregs in effecting the outcome. In contrast with wild-type (WT) mice subjected to cecal ligation and puncture (CLP) sepsis, CCR4-deficient (CCR4^-/-^) septic mice presented an increased survival rate, significant neutrophil migration toward the infection site, a low bacterial count in the peritoneum, and reduced lung inflammation and serum cytokine levels. Thus, a better early host response may favor an adequate long-term response. Consequently, the CCR4^-/-^ septic mice were not susceptible to secondary fungal infection, in contrast with the WT septic mice. Furthermore, Tregs cells from the CCR4^-/-^ septic mice showed reduced suppressive effects on neutrophil migration (both *in vivo* and *in vitro*), lymphocyte proliferation and ROS production from activated neutrophils, in contrast with what was observed for Tregs from the WT septic mice. These data show that CCR4 is involved in immunosuppression after severe sepsis and suggest that CCR4^+^ Tregs negatively modulate the short and long-term immune responses.

## Introduction

More than 750,000 cases of sepsis occur annually in the United States. Septic shock is undoubtedly the most common lethal illness, accounting for approximately 10% of intensive care unit admissions. In 2008, the mortality rates after severe sepsis and septic shock were approximately 27% and 45%, respectively [1]. Despite the availability of an increasing array of potent antibiotics and intensive medical care, sepsis mortality remains high [2].

One of the main consequences of severe sepsis is a profound state of immunosuppression, which is dependent on diverse cellular and molecular mechanisms [3]. Previous studies have shown that 80% of post-sepsis patients die within 8 years due to non-septic causes. The causes of death for these patients have included cancer, pneumonia and cardiovascular disease, which have all been correlated with the first insult [4]. In most cases, the post-sepsis state is clinically correlated with high risks of secondary infection and mortality [5,6]. The basis for this immunosuppression remains poorly understood. However, several lines of evidence have established that death from septic shock may be due to distinct mechanisms that occur over time, including enhanced leukocyte apoptosis, lymphocyte anergy, epigenetic changes and cellular reprogramming [7,8]. The latter mechanism is characterized by different activation profiles of cells such as dendritic cells [9], macrophages [10] and neutrophils [11–13] after severe sepsis. Patients after sepsis are clearly immunosuppressed, as evidenced by their development of immune anergy, frequent inability to eradicate primary infection, reduced expression of antigen-presenting molecules, shift to anti-inflammatory cytokines and toward a Th2 response and propensity to acquire secondary nosocomial infections [14].

CCR4 is a high-affinity receptor for CCL17 and CCL22 found on dendritic cells, macrophages, NK cells, platelets and basophils, but it is predominantly known for its expression on T cells, especially those with the Th2 phenotype [15]. Increased expression of CCR4 and its ligands occurs in several diseases, including pulmonary fibrosis [16,17], eosinophilic pneumonia [18], hepatic inflammation [19], granuloma development [20], and diabetes [21]. Furthermore, colon ascendens stent peritonitis (CASP)-induced sepsis in mice has been shown to increase CCL17 and CCL22 mRNA levels in the spleen and liver [22]. Additionally, a high CCL17 level has been detected in supernatant from resident peritoneal macrophages treated with LPS [23].

The majority of CCR4^+^ lymphocytes appear to be Th2-polarized CD4^+^ T cells that also exert suppressive functions via the production of immunosuppressive cytokines [24]. The chemokine receptors CCR4 and CCR8 are functionally expressed on human peripheral blood [25] and murine CD4^+^CD25^+^ T regulatory cells (Tregs) [26]. In patients with ovarian carcinoma, the host response to the tumor has been shown to be inhibited by Foxp3^+^CCR4^+^ Tregs recruited to the tumor by CCL17 and CCL22 [27]. In a mouse cardiac allotransplantation model, CCR4 and CCL22 have been shown to be up-regulated in tolerized allografts, and tolerance induction has not been achieved in CCR4-deficient (CCR4^−/−^) mice, indicating an important role of CCR4 in the generation and/or recruitment of Foxp3^+^ Tregs to cardiac allografts [28].

We have previously described post-inflammatory immunosuppression in a mouse model of sepsis induced by the cecal ligation and puncture (CLP) procedure and challenged with intratracheal injection of *Aspergillus fumigatus* (*A*. *fumigatus*) at three days after CLP surgery. Compared with the non-septic group, the post-septic mice showed a markedly impaired ability to clear the fungal infection. Even at two weeks after CLP, the mice remained susceptible to *A*. *fumigatus*, suggesting the existence of long-term immunosuppression following severe sepsis [29]. Our goal was to understand the roles of CCR4 and Tregs in the early event during severe sepsis and in the susceptibility of septic mice to a secondary infection with this fungus.

On the basis of these data, we hypothesized that CCR4^+^ cells modulate the innate immune response by promoting neutrophil migration failure, reducing neutrophil activation and enhancing the suppressive effects of Tregs during the first and second insults, thereby contributing to the outcomes of severe sepsis and immunosuppression. Our results demonstrate that CCR4 may play important roles in both the innate and acquired immune responses and suggest that the overwhelming acute response contributes to the suppressive function of Tregs.

## Materials and Methods

### Mice

Male and female wild-type C57Bl/6 (WT) and CCR4^−/−^ mice from the same genetic background, 6 to 8 weeks of age, were used in this study. The CCR4^−/−^ breeding pairs were a gift from Dr. Steven Kunkel (University of Michigan, Ann Arbor, MI, USA) and were raised in the animal facility of the Pharmacodynamics Department in FIOCRUZ, RJ, Brazil. The animals were kept at a constant temperature (25°C) under a 12 h light/dark cycle with free access to food and water. All experiments were conducted and approved in accordance with the ethical guidelines of the Institutional Animal Care Committee-CEUA in UFRJ, Rio de Janeiro, RJ, protocol code: DFBCICB028.

### CLP model

Sepsis was induced by CLP as previously described [30]. Briefly, mice were anesthetized with ketamine and xylazine (112.5 mg/kg and 7.5 mg/kg i.p., respectively), and a 1 cm midline incision was made on the anterior abdomen. The cecum was exposed and ligated below the ileocecal junction without causing total bowel obstruction. Using a 21-gauge needle, we punctured the cecum nine or three times to induce lethal sepsis (L) or non-lethal sepsis (NL), respectively. For some experiments, we refer to the CLP group as the lethal sepsis (nine punctures) group in the figures. The cecum was returned to the abdominal cavity, and the peritoneal wall and skin incision were closed with a surgical 9 mm clip. All animals received 1 mL of sterile isotonic saline s.c. immediately after surgery. Sham-operated animals (sham) underwent identical laparotomy but without cecal ligation or perforations.

The mice in both surgery groups were treated with an ertapenem antibiotic preparation (75 mg/kg i.p., Invaz, Merck) beginning at 6 h after CLP surgery and every 24 h thereafter until day 3 post-surgery. Antibiotic therapy [29,31] increased the survival of the animals by 60–70%, and the surviving animals were used in the following experiments. All animals subjected to lethal sepsis developed early clinical signs of sepsis, including lethargy, piloerection, tachypnea and an increased heart rate. The survival rate was determined daily for 7 days.

### Neutrophil migration toward peritoneal cavity and alveolar space

Cell migration to the peritoneal cavity was evaluated at 6 h after CLP. The peritoneal cavity of each animal was washed with 2 mL of a sterile saline solution containing 1 mM EDTA. Peritoneal exudates were collected for total and differential leukocyte counts.

In another set of experiments, bronchoalveolar lavage (BAL) was performed at 24 h after i.t. administration of saline, *A*. *fumigatus* conidia alone, or *A*. *fumigatus* conidia together with Tregs purified from WT and CCR4^-/-^ animals from the sham and CLP donors. Briefly, mice were euthanized in a CO_2_ chamber, the trachea was exposed and BAL was performed by instilling 1 mL of PBS. The total leukocyte count was determined with a hemocytometer under optical microscopy after diluting the samples in Türk solution (2% acetic acid plus violet blue crystal in PBS buffer). Differential leukocyte counts in peritoneal exudates and BAL samples were determined in cytospin smears stained by the Diff-Quick method. One hundred cells were counted, and the percentages of mononuclear cells, eosinophils and neutrophils were determined; however, the results were expressed only as the number of neutrophils per cavity or BAL. Cell-free supernatants of peritoneal exudate and BAL were stored at -80°C for future cytokine quantification.

### Bacterial counts in blood and peritoneal exudates

Animals were euthanized in a CO_2_ chamber, and blood was collected by cardiac puncture under sterile conditions. Peritoneal exudates were centrifuged at 450 g for 10 min at 4°C, and 20 μL aliquots of cell-free supernatant were assessed for bacterial CFUs. Ten-fold serial dilutions of peritoneal exudates and neat blood from each mouse were plated on Muller—Hinton agar dishes (Difco Laboratories, Detroit, MI) and incubated at 37°C. After 18 h, the number of colonies was determined and expressed per cavity or per mL of blood. Peritoneal exudates and blood from the sham groups were also evaluated as controls of the procedure, and no bacteria was detected in any plate (neat aliquots). Plasma was isolated from all blood samples and stored at -80°C for future cytokine quantification.

### Lung tissue myeloperoxidase (MPO) activity

Neutrophil sequestration in the lung was measured indirectly by MPO enzyme activity quantification as described previously [32]. Briefly, animals were euthanized in a CO_2_ chamber, and lung tissues (50–100 mg) were harvested and homogenized in 0.4 mL of ice-cold buffer (0.1 M NaCl, 20 mM Na-phosphate, 15 mM Na-EDTA, pH 4.7) and centrifuged at 800 g for 10 min. The pellet was then subjected to hypotonic lysis (0.2% NaCl solution) to remove erythrocytes. After centrifugation, the pellet was resuspended in 200 μL of sodium phosphate buffer (50 mM, pH 5.4) containing 0.5% H-TAB. MPO activity in the resuspended pellet was quantified spectrophotometrically (absorbance at 450 nm) using tetramethylbenzidine (1.6 mM) and H_2_O_2_ (0.5 mM) as substrates. We constructed a standard curve with known concentrations of neutrophils, which were subjected to the same procedure used for MPO activity detection, and the results were expressed as the number of neutrophils per mg of tissue.

### Cytokine and chemokine levels

The magnitude of the inflammatory response was determined by quantifying MIP-2 (data not shown), TNF-α, KC and IL-10 levels in the peritoneal exudate, BAL and plasma by ELISA. All measurements were performed in duplicate following the manufacturer's instructions (Peprotech, Rocky Hill, NJ).

### Flow cytometry

Blood samples, peritoneal cells, and some organs, such as the lungs, spleen and mesenteric lymph nodes, were collected on days 1 and/or 4 after sham or CLP surgery. Cell suspensions were blocked with purified anti-mouse CD16/CD32 (FcγIII/II receptor; 2.4G2 clone) from Pharmingen. CD4^+^Foxp3^+^ expression was analyzed by surface staining with CD4-PerCP and intracellular staining with Foxp3-FITC. Surface staining was also performed to assess the expression of CD11c^+^-PECy5, NK1.1^+^-PE, and F4/80^+^-PE in the lung cell suspension on day 1 after sham or CLP surgery. Data were obtained from gated SSC/FSC T cells. Flow cytometry analysis was performed with a FACSCalibur system (CELLQuest software; Becton and Dickinson, Mountain View, CA). Tregs were gated on CD4^+^ cells, and the percentages of the second marker were calculated. The data were expressed as the absolute cell number (tissue), the cell proportion (tissue and cavity) or as cells/ mL (blood and cavity).

### *A*. *fumigatus* culture conditions and intrapulmonary infection

To further evaluate the effects of immunosuppression following severe sepsis on the pulmonary innate immune response, we examined the impact of i.t. administration of *A*. *fumigatus* conidia as a second insult to mice previously subjected to sham or CLP surgery and antibiotic therapy. *A*. *fumigatus* (strain 13073; American Type Culture Collection, Rockville, MD) was grown on Sabouraud dextrose agar culture plates (Becton & Dickinson, Cockeysville, MD) at 37°C, and *A*. *fumigatus* conidia were harvested on day 7 of culturing. Conidia were harvested from each culture plate by washing with 50 mL PBS containing 0.1% Tween-80. The conidial suspension was passed through four layers of sterile gauze and counted using a hemocytometer. It was then diluted to the desired concentration and kept on ice until administration.

To deliver the second insult, WT and CCR4^-/-^ mice were anesthetized on day 4 after sham or CLP surgery, and a 0.5 cm incision was applied to separate the skin and muscle immediately above the trachea. Each mouse then received 7.5 x 10^7^ *A*. *fumigatus* conidia suspended in 30 μL PBS or vehicle only via the i.t. route using a 1-mL syringe controlled by a Stepper (Tridak Division, Indicon, Inc., Brookfield, CT). The incision was closed with a surgical 9 mm clip. Survival was monitored for up to 7 days. To evaluate pulmonary histology, we euthanized the mice on day 2 after the *A*. *fumigatus* conidia challenge.

### Lung histology

At two days after fungal challenge, PBS was perfused through the left ventricle in the surviving mice, followed by i.t. perfusion with PBS/OCT (2:1). The lungs were embedded in OCT and stored at -20°C. Routine histologic techniques were used, and a 5 μm section of whole lung was stained with H&E. Histology images were captured for one experiment (n = 3 per group).

### Lung morphometry

On day 5, whole lung samples were perfused with 10% formalin, placed in buffered formalin for an additional 24 h, and then processed for histological analysis. Routine histological techniques were performed using paraffin-embedded tissue, and 5 μm sections of whole lung were stained with hematoxylin-eosin (H&E). The intensity of edema in the perivascular, peribronchial, septal, and alveolar spaces and the degree of cell infiltration in the perivascular, interstitial, and alveolar spaces were examined using a previously published [33] scoring system to measure the severity of acute lung injury as follows: perivascular edema = 1; peribronchial edema = 2; interstitial edema = 2; alveolar edema = 3; perivascular cell infiltration = 2; interstitial cell infiltration = 3; and alveolar cell infiltration = 4. A total of 10 fields were examined for each lung tissue. The scores were summed for each field, and then the mean score for 10 fields was calculated, representing the injury score for each lung tissue [34].

### Isolation of Tregs

CD4^+^CD25^+^ T cells were purified from pools of murine spleens and lymph nodes. All cell populations were isolated using a biotinylated antibody cocktail followed by a streptavidin microbeads system (Miltenyi Biotec, Auburn, CA). CD4^+^ T cells were isolated by negative selection with MACS LD columns (Miltenyi Biotec) subjected to a magnetic field. CD4^+^CD25^+^ purification was performed by staining with anti-CD25-PE, followed by staining with anti-PE microbeads (positive selection). CD4^+^CD25^+^ cells were isolated using a magnetic field and were then eluted from the MS column. The purity of CD4^+^CD25^+^ T cells was greater than 85–90%, as determined by flow cytometry analysis. For the adoptive transfer of Tregs, CD4^+^CD25^+^ T cells were obtained from WT and CCR4^-/-^ mice on day 4 after sham or CLP surgery. Each mouse received i.t. co-injection of 5 x 10^7^ *A*. *fumigatus* conidia with 5 x 10^4^ Tregs purified from WT and CCR4^-/-^ animals from the sham and CLP groups. In another set of experiments, CD4^+^CD25^+^ T cells were purified from the same groups described above and incubated at a concentration of 10^6^ cells/mL overnight at 37°C. Then, the cells were removed by centrifugation, and supernatants were stored at -20°C for ROS production assay.

### Isolation of neutrophils from bone marrow

Bone marrow neutrophils were isolated as described previously [35]. Femurs and tibias of C57BL/6 mice were dissected, and bone marrow was flushed with HBSS and layered on a two-step Percoll gradient (72% and 65%), which was centrifuged at 1200 g for 32 min. Cytospin samples of the 72–65% band interface revealed the presence of >95% morphologically mature-appearing neutrophils.

### Numbers of neutrophils in blood and bone marrow

Neutrophils were isolated from bone marrow as described above. Briefly, after the Percoll gradient step, cells were collected, washed twice with PBS and counted with a hemocytometer. The data were expressed as absolute number of neutrophils per femur. The percentage of neutrophils in the blood was determined in cytospin smears stained by the Diff-Quick method, in which one hundred cells were counted. The results were expressed as the percent of neutrophils in the blood.

### Chemotaxis assay

CD4^+^CD25^+^ Tregs were obtained from WT and CCR4^-/-^ mice at four days after sham or CLP surgery. Freshly isolated neutrophils were first incubated with CD4^+^CD25^+^ Tregs (ratio 39:1) for 2 h at 37°C. Then, 2 x 10^5^ neutrophils were resuspended in 100 μL of medium (RPMI 1640 plus 1% FBS) and added to the upper wells of 24-well tissue culture inserts with 5 μm-pore size membranes (Corning, Costar, Tewksbury, MA). In the bottom wells, 1 nM of diluted LTB_4_ in medium was loaded. As positive and negative controls, the migration of neutrophils alone toward the 1 nM LTB_4_ or medium, respectively, was assessed. The cells that migrated were collected from the bottom well after a 2 h incubation at 37°C and counted using a hemocytometer.

### Reactive oxygen species (ROS) production

To evaluate whether Tregs negatively modulate neutrophil functions via a soluble factor, the Tregs supernatants were collected as described above, diluted 1:20 and incubated with 2 x 10^5^ freshly isolated neutrophils pooled from 3–4 naïve mice for 2 h at 37°C. Then, the neutrophils were stimulated with 50 ng/mL of PMA or RPMI (control) for 1 h. They were next incubated with a ROS probe (2 μM ROS Detection Probes; CM-H_2_DCFDA, 5-(and-6)-chloromethyl-2',7'-dichlorodihydrofluorescein diacetate, acetyl ester; Invitrogen, Grand Island, NY) and maintained at 37°C in the dark for 10 min. Then, the cells were pelleted and resuspended in PBS for flow cytometry analysis. The data were expressed as the mean fluorescence intensity (MFI) determined from one experiment, representative of two independent experiments.

### Carboxyfluorescein succinimidyl ester (CFSE) staining and proliferation assay

Tregs were obtained from WT and CCR4^-/-^ septic animals at 4 days after CLP surgery. For CFSE labeling, splenocytes obtained from naïve mice were stained with CFSE (1.5 μL 10 mM for 5 x 10^6^ cell/mL) for 15 min at 37°C and washed 3 times with RPMI 1640 containing 10% FCS. The plate was pre-coated overnight with anti-CD3 (5 μg/mL). Purified Tregs were incubated with CFSE-splenocytes (1:4 ratio) for 96 h at 37°C in the presence of anti-CD28 (2 μg/mL). Cells were recovered and stained with anti-CD4 PE-Cy5. Proliferation was evaluated by flow cytometry.

### Statistical analysis

Survival curves were generated with GraphPad Prism software (Graphpad Software, Inc., San Diego, CA), and comparisons between the curves were made using the log-rank test. All other data were expressed as the mean ± SEM and compared using the Mann-Whitney test. Data were considered statistically significant at a *P* value of less than 0.05.

## Results

### CCR4^-/-^ mice are more resistant to sepsis

**Fig 1:**
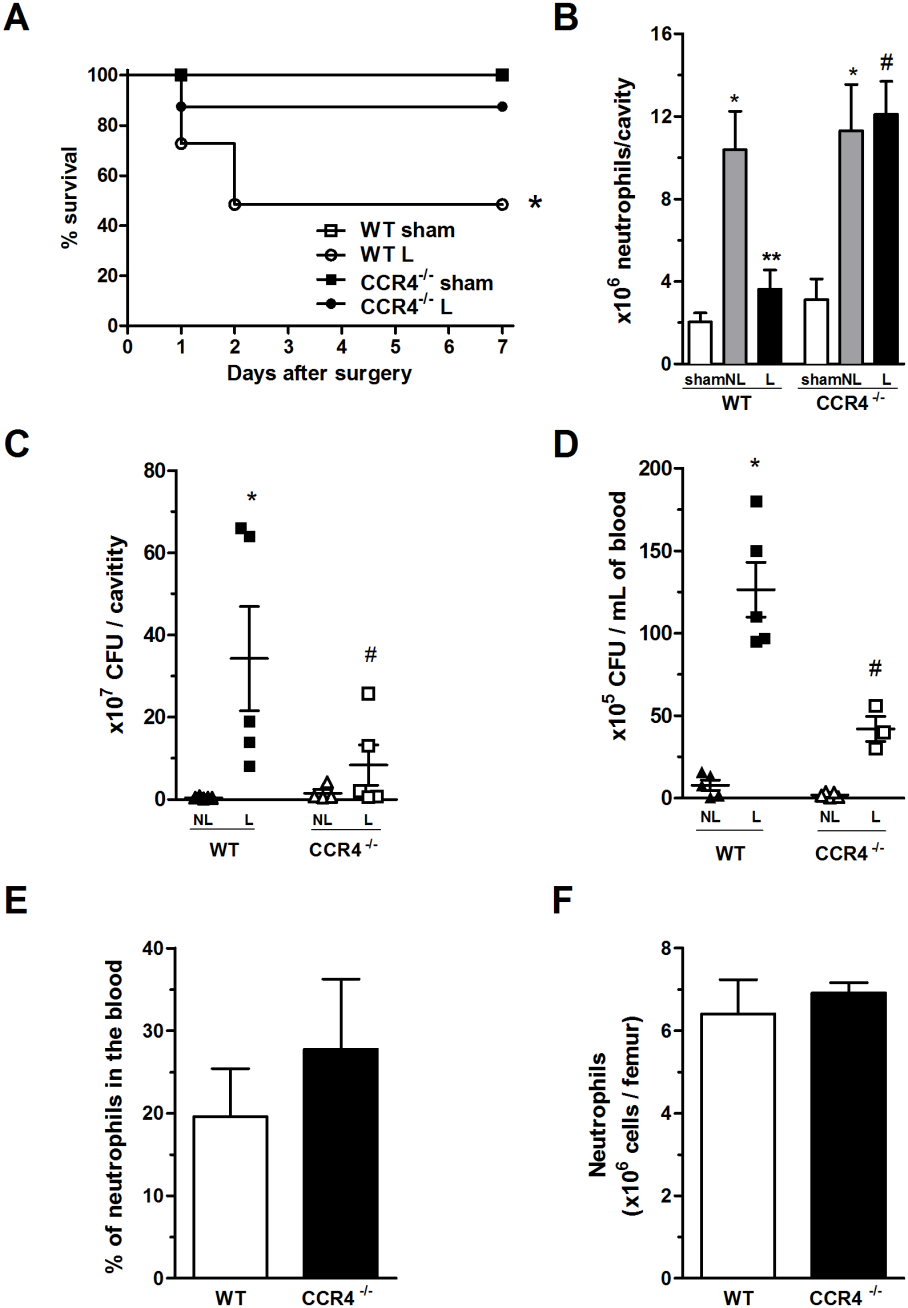
CCR4^-/-^ mice show increased resistance to severe sepsis. (A) WT (open symbols) and CCR4^-/-^ (filled symbols) mice underwent sham (square) or L-CLP (circle) surgery. All groups received antibiotic therapy of ertapenem (75 mg/kg) at 6, 24 and 48 h after surgery. The survival of these mice was followed until day 7. The data are representative of four experiments (n = 7–11 mice per group). *P<0.05 between the CLP CCR4^-/-^ and CLP WT groups. (B) Neutrophil migration to the peritoneal cavity in the sham, Non-Lethal CLP (NL) and Lethal-CLP (L) groups evaluated at 6 h after surgery. *P<0.05 between the NL-CLP and sham groups of both strains (WT and CCR4^-/-^); **P<0.05 between L-CLP and NL-CLP groups of WT mice; and #*P*<0.05 between the L-CLP WT and L-CLP CCR4^-/-^ mice. (C) Bacterial counts in peritoneal exudate and (D) blood at 6 h after NL- or L-CLP surgery of the WT and CCR4^-/-^ mice. *P<0.05 between the L-CLP WT and NL-CLP WT groups; and #*P*<0.05 between the L-CLP CCR4^-/-^ and L-CLP WT groups. (E) Percentages of neutrophils in the blood of naïve WT and CCR4^-/-^ mice. (F) Absolute numbers of neutrophils in the bone marrow of naïve WT and CCR4^-/-^ mice.

To characterize the role of the CC chemokine receptor CCR4 in polymicrobial severe sepsis, we performed CLP surgery, as described by Benjamim *et al*. [29], on WT and CCR4^-/-^ mice. Both groups of mice received antibiotic treatment for 3 days. The absence of CCR4 receptor significantly increased the survival rate at 7 days after CLP (Fig 1A). Septic CCR4^-/-^ mice (L-CCR4^-/-^) had a low mortality rate (10%), whereas 52% of the septic WT mice (L-WT) died. All WT and CCR4^-/-^ mice subjected to sham surgery survived. This finding confirms those of previous studies, indicating that CCR4^-/-^ mice without antibiotic treatment are more resistant to high and low doses of LPS [15], TLR2 agonists [23], and mild septic peritonitis [10].

### CCR4^-/-^ mice exhibit neutrophil migration and low bacterial counts

**Fig 2:**
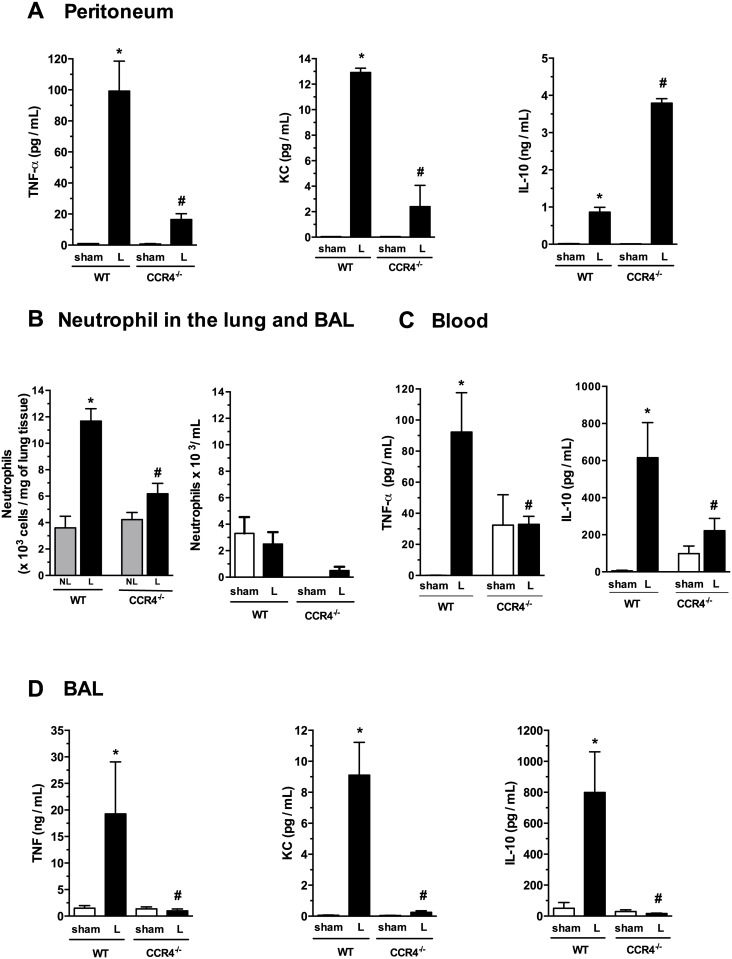
L-CCR4^-/-^ mice have reductions in TNF-α and KC levels and an increased production of IL-10 in the peritoneal cavity and do not develop a systemic inflammatory response. (A) TNF-α, KC and IL-10 levels were detected in peritoneal exudates at 6 h after surgery by ELISA, as described in the Methods section. *P<0.05 between the L-WT and sham-WT groups; and #*P*<0.05 between the L-CCR4^-/-^ and L-WT groups. (B) Absolute numbers of neutrophils were determined in the lung tissue (left panel) and BAL (right panel) at 6 h after surgery by MPO assay or cell counts, respectively. *P<0.05 between the L-WT and NL-WT groups; and #*P*<0.05 between the L-CCR4^-/-^ and L-WT groups. (C) TNF-α and IL-10 levels were detected at 6 h after surgery in blood from WT and CCR4^-/-^ mice in the sham and L-groups. *P<0.05 between the L-WT and NL-WT groups; and #*P*<0.05 between the L-CCR4^-/-^ and L-WT groups. (D) TNF-α, KC and IL-10 levels were detected at 6 h after surgery in BAL by ELISA. *P<0.05 between the L-WT and sham-WT groups; and #*P*<0,05 between the L-CCR4^-/-^ and L-WT groups.

It is well known that during severe sepsis, neutrophil migration to the infection site fails, and as a consequence, a bacterial burst occurs at the site of injury (the peritoneal cavity in the case of the CLP sepsis model) and in the blood [13]. Therefore, we confirmed that the L-WT mice had reduced neutrophil migration toward the peritoneal cavity at 6 h after CLP, while interestingly, the L-CCR4^-/-^ group did not exhibit the failure of neutrophil migration (Fig 2B). Such neutrophil accumulation was similar to that observed in WT and CCR4^-/-^ mice subjected to non-lethal sepsis (NL-CCR4^-/-^), in which no migration failure was observed (Fig 2B). As a consequence of these findings, the L-CCR4^-/-^ mice showed reductions in CFUs in the peritoneal cavity and blood compared with those in the peritoneal cavity and blood of the L-WT mice (Fig 2C and 2D; respectively). Neutrophil recruitment and CFUs were also assessed in the WT and CCR4^-/-^ animals in the NL groups to determine the expected migration during peritonitis and bacterial counts as two sepsis severity parameters for comparisons. These results suggest that CCR4^-/-^ mice have a better innate immune response during severe sepsis than WT mice.

The results obtained for the CCR4^-/-^ mice were not due to differences in the numbers of neutrophils observed in the leukogram or in the bone marrow compared with those in the WT mice (Fig 1D and 1E, respectively), supporting the hypothesis that CCR4^-/-^ mice have a more adequate and intense innate response in terms of neutrophil migration and bacterial clearance than WT mice.

### L-CCR4^-/-^ mice present an increased production of IL-10 and reductions in TNF-α and KC levels in the peritoneal cavity

To further investigate the role of cytokines at the infection site, mice were subjected to sham or CLP surgery, and 6 h later, the presence of cytokines in peritoneal exudates were analyzed. Fig 2A shows that TNF-α (left panel), KC (middle panel), and IL-10 levels (right panel) were higher in the L-group in both the WT and CCR4^-/-^ mice compared with the respective sham groups. Interestingly, the TNF-α and KC levels were lower in the L-CCR4^-/-^ mice compared with the L-WT mice, while the IL-10 level was significantly higher compared with the L-WT mice. The low levels of pro-inflammatory mediators and high level of IL-10 reflect the better evolution of the immune response.

### CCR4^-/-^ mice have a reduced systemic inflammatory response

Next, we determined whether CCR4^-/-^ mice produce a systemic inflammatory response by evaluating neutrophil infiltration in the lung, a secondary target organ, at 6 h after surgery. MPO assay showed that the L-CCR4^-/-^ mice had a lower level of neutrophil accumulation in pulmonary tissue compared with the L-WT mice (Fig 2B, left panel). The NL-WT and NL-CCR4^-/-^ mice presented the same MPO activity levels. The NL groups was used here as a control because the sham group did not have detectable MPO activity in lung tissue due to the lack of resident neutrophils. Moreover, we were unable to detect relevant neutrophil recruitment to the BAL in either the WT or CCR4^-/-^ mice in the sham or L- group at the time point evaluated (Fig 2B, right panel). Furthermore, with regard to systemic cytokines, the L-CCR4^-/-^ mice had lower TNF-α and IL-10 plasma levels compared with the L-WT mice (Fig 2C). Similarly, the TNF-α, KC, and IL-10 levels in the BAL of the L-CCR4^-/-^ mice were reduced compared with those in the L-WT mice (Fig 2D). In addition, we determined CFU counts in the BAL of the L-WT and L-CCR4^-/-^ mice and found that the latter group had no bacteria in the cavity, while the L-WT mice presented ~1300 CFU/cavity (data not shown). These results together indicate a better local response, and consequently, a less compromised systemic immune response in the CCR4^-/-^ mice subjected to CLP surgery.

### Tregs numbers in WT and CCR4^-/-^ mice after CLP surgery

**Fig 3:**
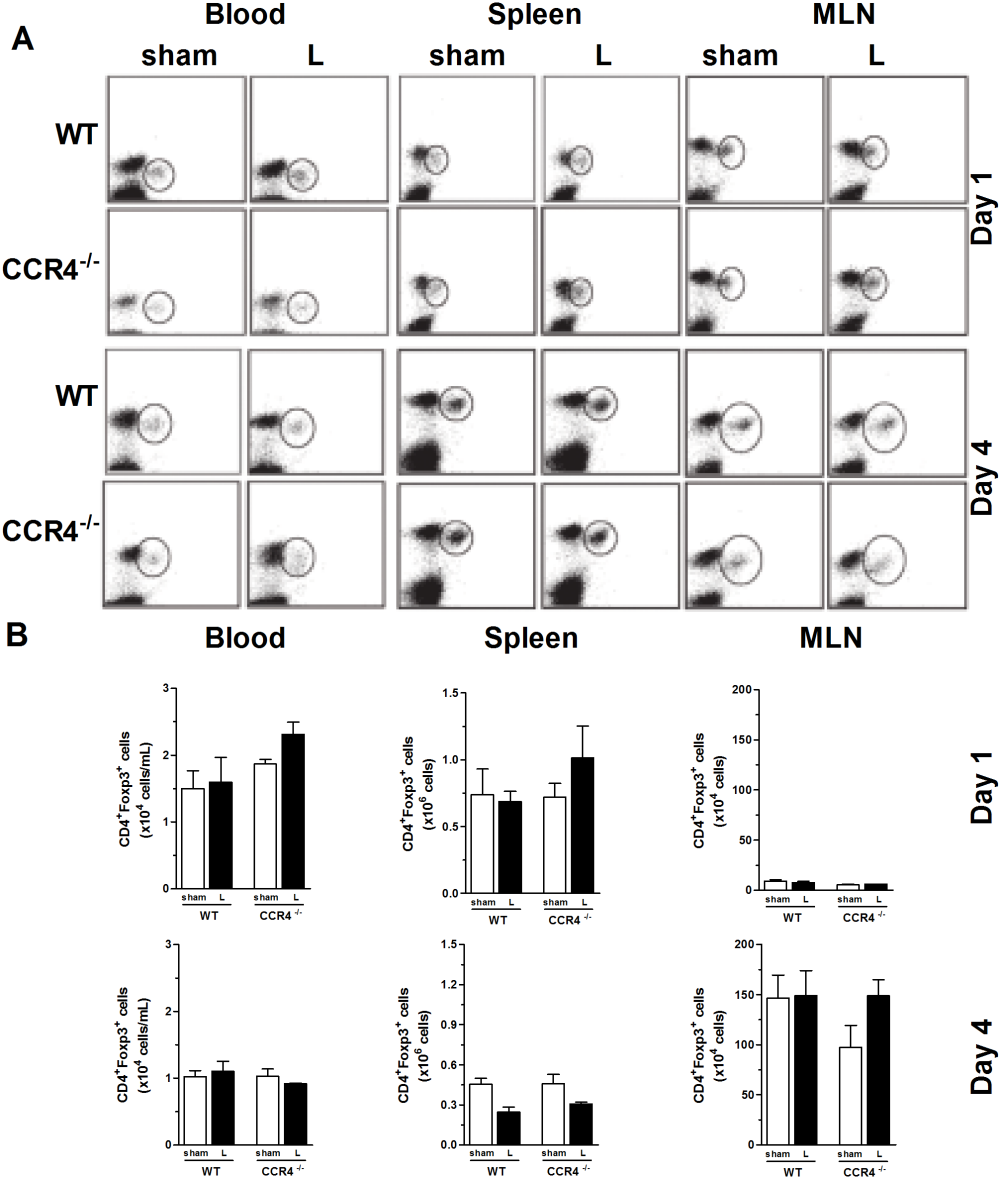
Presence of CD4+Foxp3+ Tregs in lymphoid tissue after surgery. WT and CCR4^-/-^ mice were subjected to sham or CLP surgery and antibiotic treatment with ertapenem (75 mg/kg) at 6, 24 and 48 h after surgery. On day 1 or 4 after surgery, mice were sacrificed, and blood (left two columns), spleen (middle two columns) and mesenteric lymph node (right two columns) samples were collected and fixed. The samples were stained for CD4 and Foxp3 and analyzed by flow cytometry. (A) The dot plot data show one representative mouse each from the WT and CCR4^-/-^ sham and L-groups. (B) Absolute numbers of CD4^+^Foxp3^+^ Tregs in the blood, spleen and MLN on days 1 and 4 after surgery. The graphs display the mean ± SEM for each group (n = 3–4 per group).

To understand the mechanisms involved in the resistance of CCR4^-/-^ mice to CLP, we investigated the presence of Tregs (a modulatory cell subset of the innate and acquired immune responses; CD4^+^Foxp3^+^) in the blood, spleen, and mesenteric lymph nodes (LNs). As shown in Fig 3, on days 1 and 4 after surgery, we did not detect any differences in the dot plots (panel A) or in the absolute numbers (panel B) of Tregs in the blood, spleen, or lymph nodes between the WT and CCR4^-/-^ mice. We also evaluated the number of Tregs in the lungs of the CLP WT and CCR4^-/-^ mice and observed no differences, even between the sham and CLP groups of both strains (data not shown).

**Fig 4:**
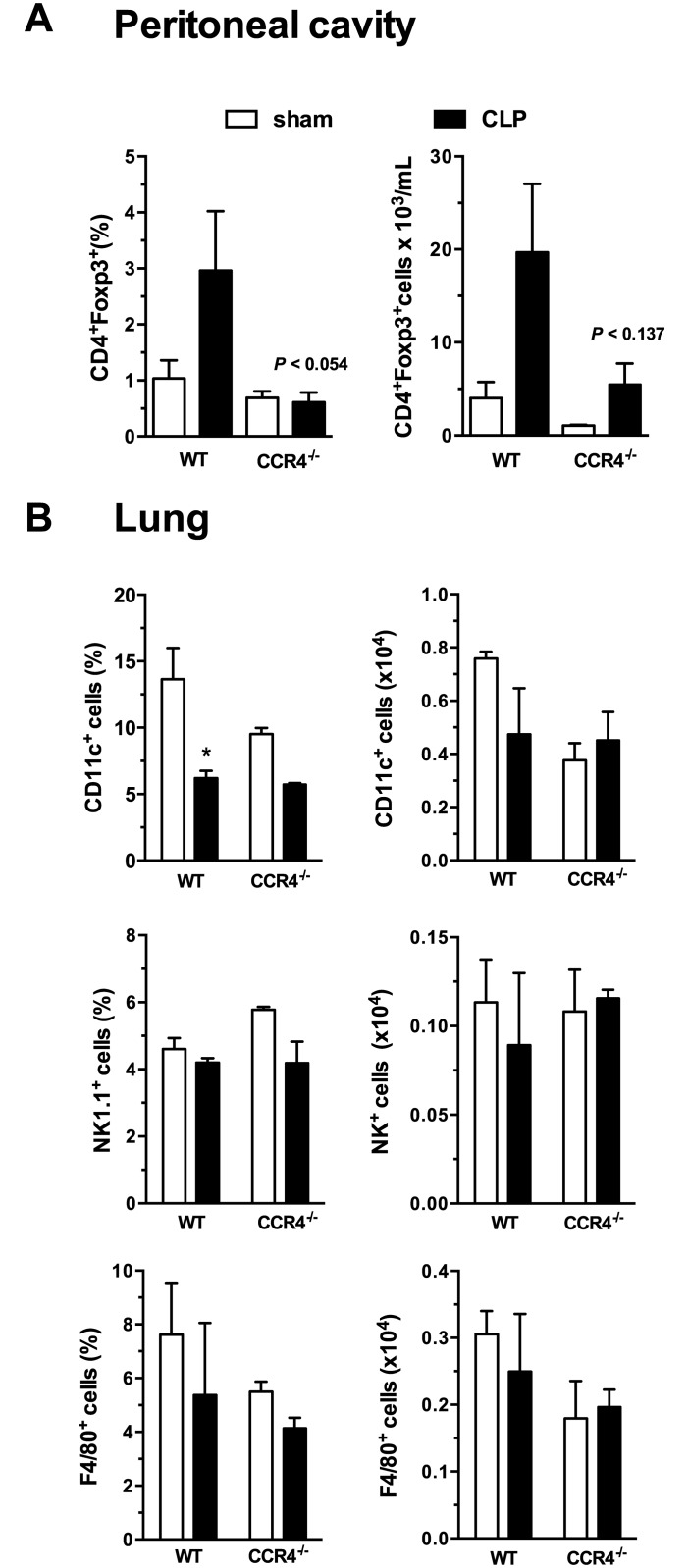
CD4^+^Foxp3^+^ Tregs in the peritoneal cavity and dendritic cells, NK cells, and macrophages in the lungs of WT and CCR4^-/-^ mice. (A) Percentages of CD4^+^Foxp3^+^ Tregs (left panel) and their absolute numbers (right panel) in the peritoneal cavity at 24 h after sham and CLP surgery in the WT and CCR4^-/-^ mice. (B) The percentages of CD11c^+^ (dendritic cells), NK1.1^+^ (natural killer cells), and F4/80^+^ cells (macrophages) (left panels) and their absolute numbers (right panels) were determined in the lungs of the WT and CCR4^-/-^ mice at 24 h after sham and CLP surgery. The data are representative of two experiments. *P<0.05 between the L-WT and sham-WT groups.

The percentages and absolute numbers of CD11c^+^ (dendritic cells), NK1.1^+^ (natural killer cells), and F4/80^+^ cells (macrophages) were also investigated in the lungs after sham and CLP surgery. We observed a significant reduction in the percentage of dendritic cells (from ~14% to 4% CD11c^+^ cells) in the L-WT group compared with the sham WT group, which was expected (Fig 4B) because a reduction in DCs after severe sepsis has already been reported by our group and others [9]. This result was not observed for the L-CCR4^-/-^ group. Moreover, no differences (even in the cell percentages and absolute numbers) were observed for the F4/80^+^ and NK1.1^+^ cells in the lungs of the L-CCR4^-/-^ mice compared with those in the lungs of the other groups of mice (Fig 4B).

In addition, we evaluated the Tregs population in the peritoneal cavity at 1 day after sham and CLP surgery. Despite the fact that the differences in Tregs numbers in the peritoneal cavity between the groups did not reach statistical significance, it is important to highlight that there was a tendency of an increase in the Tregs number in the L-WT group compared with the sham-WT group, while such an increase was not observed in the L-CCR4^-/-^ group (Fig 4A).

### CCR4^-/-^ mice are protected against secondary infection

**Fig 5:**
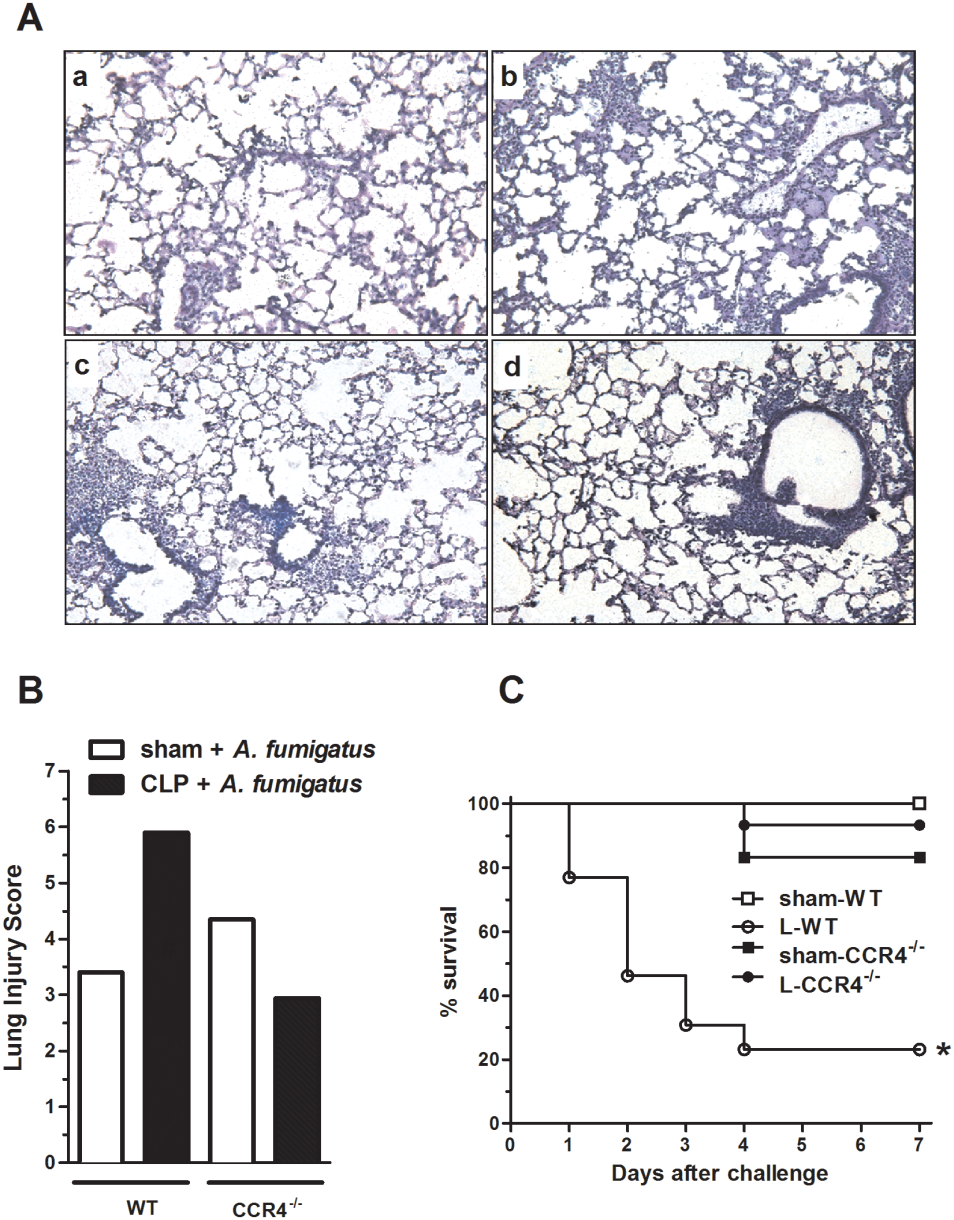
CCR4^-/-^ post-septic mice present a better survival rate and reduced lung damage after *A*. *fumigatus* challenge. The WT and CCR4^-/-^ mice were subjected to sham or CLP surgery and received antibiotic treatment with ertapenem (75 mg/kg) at 6, 24 and 48 h after surgery. On day 4 after surgery, the surviving CLP and sham mice were challenged with 7.5 x 10^7^ of *A*. *fumigatus* conidia i.t. (60–70% of the survivors after CLP surgery represented 100% of the mice at time 0). (A) On day 4 after surgery, the sham-WT, L-WT, sham-CCR4^-/-^, and L-CCR4^-/-^ mice were challenged *with A*. *fumigatus*, and after 48 h, all mice were euthanized, and their lungs were collected for histology. The lungs were fixed, and 5-μm sections were stained with H&E. (B) Histological appearances were scored for perivascular, peribronchial, septal, and alveolar edema and for perivascular, interstitial, and alveolar cell infiltration. The pathological changes differed significantly between the L-WT and L-CCR4^-/-^ groups. (C) Survival was followed until 7 days after fungal challenge. The data are representative of two experiments; n = 9–15 mice per group. *P<0.0001 compared with the L-WT group.

Because CCR4^-/-^ mice were more resistant against septic insult, we hypothesized that these animals may also exhibit a higher resistance to a secondary infection challenge. To investigate this hypothesis, we designed an experimental protocol in which mice that survived to CLP (after antibiotic treatment) were i.t. challenged with *A*. *fumigatus* conidia on day 4 after surgery. As illustrated in Fig 5, panels A and B, histological analysis of lungs obtained from the L-CCR4^-/-^ group mice subjected to *A*. *fumigatus* challenge revealed slight reductions in cellular infiltration, edema and conserved alveolar septa compared with lungs from the L-WT mice. WT and CCR4^-/-^ mice that were subjected to sham surgery presented similar features of pulmonary injury after fungal challenge. Similarly, the *A*. *fumigatus* conidia challenge did not cause mortality in the sham-WT mice; however, the same fungal challenge resulted in the death of 75% of the L-WT animals. Conversely, under the same conditions, the CCR4^-/-^ mice subjected to CLP presented only an approximately 5% mortality rate, demonstrating that these mice were also more resistant to the secondary insult (Fig 5C).

These data reinforce our hypothesis that the efficient innate immune response of CCR4^-/-^ mice leads to the rapid elimination of bacteria from the infectious site (peritoneal cavity), resulting in less dissemination and systemic effects/consequences.

### Tregs from L-CCR4^-/-^ mice do not exhibit suppressive activity

**Fig 6:**
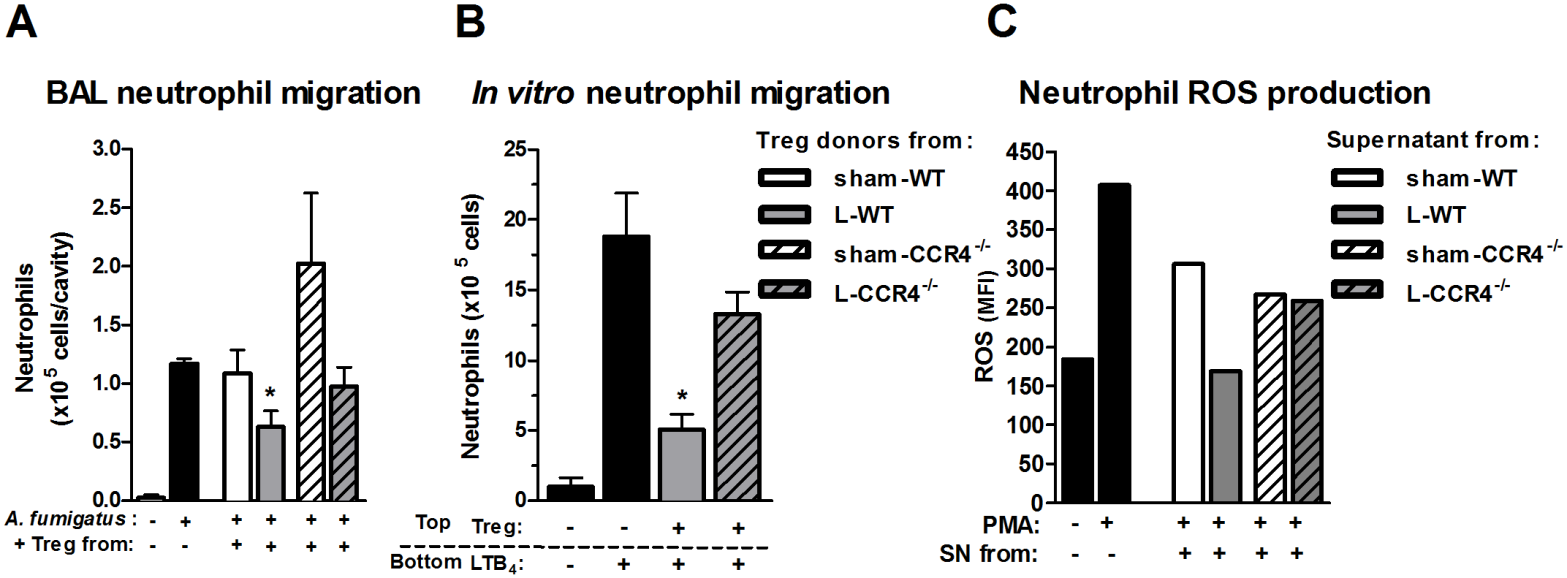
Tregs from CCR4^-/-^ post-septic mice exhibit attenuated suppressive activity. (A) WT naïve mice (n = 5–6 mouse per group) were challenged with 5 x 10^7^ *A*. *fumigatus* conidia or saline (SAL) or co-injected with 5 x 10^4^ of CD4^+^CD25^+^ Tregs obtained from WT (sham or L) or CCR4^-/-^ mice (sham or L). Tregs were purified from spleens and mesenteric lymph nodes at 4 days after surgery and were then used for co-injection. After 24 h, BAL was performed to evaluate neutrophil population/migration. The results are expressed as the number of neutrophils in the BAL. * *P*<0.05 compared to the *A*. *fumigatus* challenged group (second bar) and to the sham-WT group. (B) Bone marrow-derived neutrophils were pre-incubated with CD4^+^CD25^+^ Tregs (ratio 39:1) obtained from L-WT or L-CCR4^-/-^ mice for 2 h at 37°C on day 4 after surgery. After pre incubation, the cells were incubated in 24-transwell plates for 2 h at 37°C. Neutrophil migration was evaluated by cell accumulation in the bottom chamber. *P<0.05 compared with the group challenged with conidia that received Tregs from WT-sham group. (C) Bone marrow-derived neutrophils were incubated with supernatants (1:20) from WT CD4^+^CD25^+^ Tregs (sham or L) or CCR4^-/-^ CD4^+^CD25^+^ Tregs (sham or L) for 2 h at 37°C. After incubation, the cells were stimulated with PMA or PBS for 1 h. They were then centrifuged, and a ROS detection probe (CM-H_2_DCFDA) was used to measure ROS production by flow cytometry. The results are expressed as MFI. The data are representative of two experiments, and each experiment was performed with neutrophil pools from 3–4 mice. Statistical analysis was not conducted because the data were generated from pooled cells.

To evaluate the suppressive activity of Tregs in our model, we purified them from WT and CCR4^-/-^ lymphoid organs on day 4 after sham or CLP surgery. Then, they were co-injected with *A*. *fumigatus* i.t. into naïve animals, and 24 h later, neutrophil migration was evaluated. Interestingly, Tregs isolated from the L-CCR4^-/-^ mice did not reduce the migration of neutrophils to BAL in response to *A*. *fumigatus*, while those isolated from the L-WT mice showed a suppressive capacity, reducing neutrophil migration toward *A*. *fumigatus* infection (Fig 6A).

The same feature was observed for neutrophil migration *in vitro*. When we incubated Tregs obtained from the L-CCR4^-/-^ mice with naïve neutrophils, no inhibition of neutrophil migration toward LTB_4_ was noted. Tregs isolated from the L-WT mice were able to inhibit neutrophil chemotaxis, as previously demonstrated (Fig 6B; [36]). These data suggested that the CCR4^-/-^ Tregs did not present suppressive activity, at least reflected by the neutrophil migration results.

### CCR4^-/-^ Tregs do not inhibit ROS production by activated neutrophils

To determine whether Tregs function by releasing soluble factors, we collected supernatants from Tregs isolated from WT and CCR4^-/-^ mice at 4 days after sham and CLP surgery. Naïve neutrophils were incubated with the supernatants to evaluate the inhibition of PMA-induced ROS production. Fig 6C shows that the supernatant from the L-CCR4^-/-^ Tregs did not inhibit PMA-induced ROS production, whereas that from the L-WT Tregs showed inhibitory activity. Therefore, these data suggest that CCR4^-/-^ Tregs from septic mice do not possess the same regulatory activity profile as that of WT Tregs from septic mice.

### CCR4^-/-^ Tregs have less suppressive effects on T effector cell proliferation

**Fig 7:**
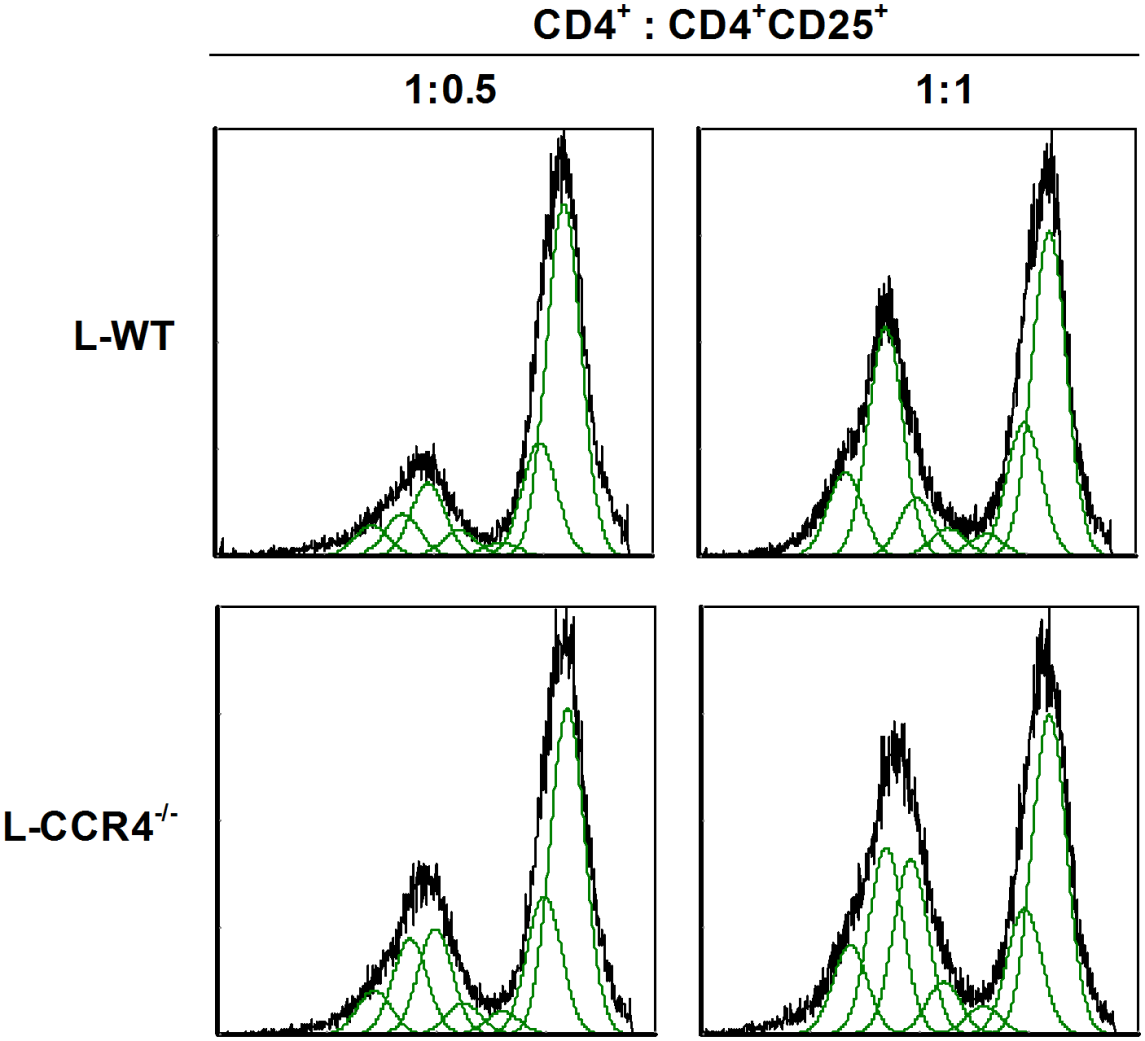
CCR4^-/-^ post-septic Tregs showed reduced suppressive effects on T effector cell proliferation. WT and CCR4^-/-^ mice were subjected to sham or CLP surgery, and on day 4 after surgery, the spleen and mesenteric lymph nodes were collected for Tregs purification. Splenocytes from naïve mice were stained with CFSE for proliferation assay. Tregs and splenocytes were co-incubated (1:4) for 96 h in the presence of anti-CD3 and anti-CD28, and the cells were analyzed by flow cytometry. These data are representative of two different experiments (n = 3 for each experiment).

T cell proliferation inhibition is a remarkable indicator of Tregs activity. Fig 7 shows that Tregs obtained from the L-CCR4^-/-^ mice presented slightly inhibited T effector cell proliferation compared with those recovered from the L-WT mice. These data support the differing activation status of the CCR4^-/-^ Tregs compared with WT Tregs.

## Discussion

Initially, sepsis was defined by the American College of Chest Physicians/Society of Critical Care Medicine consensus as a “systemic inflammatory response syndrome due to infection” based on signs observed over a period of hours to days. Currently, it is considered that cellular reprogramming, which is described as alterations in a specific set of cells, accounts for the long-term consequences of severe sepsis and septic shock, culminating in clinical signs observed over a period of months to years. The main consequences of severe sepsis are increased susceptibilities to acquired secondary infections, cancer and cardiovascular diseases [2,4,7,37]. Additionally, we believe that post-sepsis immune dysfunction may be a consequence of an initial overwhelming immune response.

Briefly, the initial inflammatory stage of sepsis is characterized by the presence of proinflammatory molecules, such as cytokines, chemokines, and lipid mediators, as well as other mediators. This initial phase is rapidly counterbalanced by an anti-inflammatory response that may adversely affect immune function. This immunosuppressed state is characterized by monocytic deactivation, manifested by decreases in phagocytic function, proinflammatory cytokine release, and antigen-presenting capacity [38,39], in addition to increases in CD8^+^, CD4^+^, and DC and NK cell apoptosis [40–42]. Moreover, several studies have reported that these inhibitory responses may hinder the host resistance to secondary nosocomial infections, thereby deleteriously affecting patient outcome.

In our first set of experiments, we demonstrated that CCR4, the receptor for CCL17 and CCL22, contributes to the establishment of an inadequate innate immune response during severe sepsis. This receptor is strongly expressed in Tregs and plays an important role in their activation and migration. This fact supports our work in using CCR4^-/-^ mice to not only investigate the effects of this receptor but also the effects of Tregs during severe sepsis. Our data demonstrated that CCR4^-/-^ mice subjected to severe sepsis presented significant neutrophil recruitment to infected sites and low bacterial counts similar to those observed in the mice with mild sepsis, which adequately responded to infection with massive neutrophil migration and consequent control of bacterial growth and high survival rates. The higher IL-10 and lower TNF-α levels detected in the peritoneal cavities of the L-CCR4^-/-^ mice also corroborated the better local response. Concerning the possible sources of IL-10 detected in the peritoneal cavities of the L-CCR4^-/-^ mice, in which we observed a reduced number of Tregs, we would like to highlight that there are several other IL-10-producing cells in the peritoneal cavity, including peritoneal neutrophils, macrophages and B-1a cells [43–45]. Therefore, it is important to state that in our model, we believe that macrophages are the major source of IL-10 in the peritoneal cavity. The L-WT mice were not able to maintain control of the infection, and even with antibiotic treatment, they had a high mortality rate (40%). The reduction of neutrophil migration toward the infection site in the experimental sepsis model has been well established [13,46–48], and this feature contributes to the dissemination of bacteria throughout the body. The mechanisms of neutrophil migration failure, which have been partly described, include an increase in NO production, a reduction in the rolling and adhesion of neutrophils onto endothelial cells, and a decrease in CXCR2 on neutrophil membranes [48,49]. This last effect is due to the activation of G protein-coupled receptor kinase 1 (GRK), which phosphorylates chemokine receptors, causing their desensitization [46–48]. The fact that the CCR4^-/-^ mice did not present with failure of neutrophil migration to the infected site suggests that this receptor plays a critical role in the innate immune response, probably through the recruitment and/or activation of immune regulatory cells during early sepsis because it is mainly present on Tregs and Th2 cells [24,25]. However, the real role of CCR4 in this early event is not completely understood. The evaluation of systemic parameters also revealed that the L-CCR4^-/-^ mice were protected because they showed reductions in neutrophil recruitment to pulmonary tissue and cytokine levels in the blood, which are both signs of systemic commitment. These data suggest that CCR4^-/-^ mice are more responsive to a threat of local infection. Using a complex animal model of polymicrobial abdominal sepsis (CLP), our results corroborate with those reported in the literature using different models, such as endotoxemia and CASP, which have also demonstrated a more effective innate immune response in CCR4^-/-^ mice [15,22,23]. Thus, the mechanism of action of CCR4 receptor in sepsis evolution is still not clear.

Patient survival immediately after a major injury or inflammatory illness has been greatly improved by advances in life support strategies; however, secondary infection is a major cause of morbidity and mortality in these patients. Interestingly, studies that have been conducted for over a decade have provided strong evidence of the role of CD4^+^Foxp3^+^ cells, a regulatory/suppressor T cell population, in a wide assortment of biological processes, including transplantation tolerance, infection, inflammation/injury and tumor persistence [50–53]. Despite the evidence that Tregs play critical roles in tolerance to self-antigens and the suppression of autoimmune reactions, relatively little is known about their importance in the development and/or maintenance of immunosuppression after sepsis. Tregs have been detected in the blood and spleen of patients and animals during the acute phase of sepsis [54–57]. An increase in the relative number of Tregs during sepsis was first reported by Monneret *et al*. in 2003 [54]. However, the absence of an effect of anti-CD25 treatment led some investigators to question the importance of Tregs during sepsis [56,58]. There is currently controversy with regard to whether the outcome of sepsis is partly dependent on Tregs and their suppressive activity [59]. Several studies have not been able to link the increase in the suppressive function of Tregs with the increase in mortality during sepsis [55,56]. Therefore, we have recently demonstrated that Tregs play a pivotal role in the triggering of long-lasting immunosuppression induced by severe sepsis. The depletion of Tregs in surviving septic mice reestablishes the immune status, and these mice become resistant to secondary nosocomial infections [60].

In this study, we assessed whether Tregs participate in the dysfunction of the acute and long-term immune responses after severe sepsis. Tregs bearing CCR4 have an exceedingly detrimental role in the CLP model [10], although it has not been described in the context of immunosuppression following sepsis until now. Our work on Tregs and CCR4 contributes to the understanding of the long-term consequences of severe sepsis. Interestingly, and in accordance with our initial data, L-CCR4^-/-^ mice were also more resistant against secondary fungal infection. Therefore, an initial insult and the presence of CCR4 on leukocyte surfaces were critical for the subsequent immunosuppression. To explain the susceptibility as a consequence of increased Tregs in lymphoid organs, as suggested by Traeger *et al*. [22], we examined the Tregs populations in the spleen, mesenteric lymph nodes and blood. At both time points analyzed, we did not detect significant differences in the number of Tregs between the WT and CCR4^-/-^ mice. Actually, on the first day after sepsis, we observed a tendency for the number of Tregs to increase in the spleen and blood of the L-CCR4^-/-^ mice. Additionally, the number of these cells seemed to increase after *A*. *fumigatus* challenge in the lungs of the L-CCR4^-/-^ mice (L-CCR4^-/-^ = 7.3% vs. infected L-CCR4^-/-^ = 13.8%), while in the lungs of the L-WT mice, the number of Tregs seemed to decrease (L-WT = 13.5% vs. infected L-WT = 10%). Initially, these results seemed contradictory because we expected a reduced number of Tregs in the secondary local infection in the CCR4^-/-^ mice due to the importance of CCR4, among other receptors, for Tregs migration. Thus, we investigated Tregs activity to clarify our findings. Our hypothesis was that the suppressive activity of Tregs in the lungs and/or lymphoid organs may be altered in the absence of CCR4.

It is already known that Tregs modulate cellular activation and migration [61]. Baatar *et al*. [62] have demonstrated that in contrast with CCR4^-^ Tregs, which have to be stimulated with anti-CD3, human CCR4^+^ Tregs appear to be already primed for regulatory activity. Additionally, CCR4^+^ Tregs produce more IL-10 and impair Th1 polarization, suggesting a relevant role of CCR4 in the suppressive function of these cells [62]. We further analyzed the potential modulatory effects of Tregs from the WT and CCR4^-/-^ mice on neutrophil migration (in response to *A*. *fumigatus* lung challenge) and on PMA-induced ROS production by neutrophils. Surprisingly and interestingly, CCR4^-/-^ Tregs from the septic mice did not suppress neutrophil migration like the WT Tregs from the septic mice *in vivo* or *in vitro*. It is important to highlight that neutrophil recruitment is a hallmark of the innate immune response. Furthermore, neutrophils incubated with supernatant from L-CCR4^-/-^ Tregs were barely inhibited for ROS production compared with those incubated with supernatant from L-WT Tregs. Notably, we were not able to statistically analyze these data because we used a pool of neutrophils obtained from three to four mice for each group; thus, the data presented is representative of two independent experiments. However, we can at the least suggest that the ROS data corroborates with the neutrophil migration and innate response data, which showed an effective cell response in the CCR4^-/-^ septic mice compared with the WT septic mice. The same response was observed in proliferation assay, which revealed that L-CCR4^-/-^ Tregs had less of an inhibitory effect on T effector cell proliferation compared with L-WT Tregs. These results suggest that even during the innate response to secondary infection, Tregs seem to negatively modulate the early response via neutrophil migration and activation.

Taken together, our data may indicate a new approach for improving the innate response against infection and impairing long-term cellular reprogramming following severe sepsis. Our data highlight the modulatory pathway, which functions through a chemokine receptor, CCR4.

Our hypothesis is that the combined use of a CCR4 antagonist with antibiotic therapy may increase the survival rate after an initial insult and also guarantee long-term survival without immunosuppression, decreasing susceptibility to pulmonary infections, cancers, and cardiovascular diseases. Although we do not have any data at the moment in support of this speculation, these experiments are underway. In summary, our work strongly suggests that during a systemic inflammatory disease such as sepsis, an early and overwhelming immune response is crucial to induce the reprogramming of neutrophils [13], dendritic cells [9] and Tregs. In addition, we suggest that CCR4 on Tregs modulates their suppressive effects and may act as a link between the innate response and long-term consequences of severe sepsis.
